# Sources of information on Gestational Diabetes Mellitus, satisfaction with diagnostic process and information provision

**DOI:** 10.1186/s12884-016-1067-9

**Published:** 2016-09-29

**Authors:** Padaphet Sayakhot, Mary Carolan-Olah

**Affiliations:** St Albans Campus, College of Health and Biomedicine, Victoria University, Building 4C, McKechnie Street, St Albans, VIC 3021 Australia

**Keywords:** Gestational Diabetes Mellitus, Satisfaction, Diagnostic process, Sources of information, Information provision

## Abstract

**Background:**

This study aimed to investigate the percentage of the needs and expectations of pregnant women with Gestational Diabetes Mellitus (GDM) about the best sources of information on GDM, their satisfaction with the diagnostic process and information provision.

**Methods:**

Questionnaires were completed by 116 pregnant women aged 18–45 years, diagnosed with GDM and recruited from maternity diabetes clinic. Eligible women were invited to participate in the study and informed consent was obtained from each participant prior to enrolment. Descriptive statistics, Kruskal-Wallis test, *t*-test and chi-square test were used to analyse data.

**Results:**

Most women (64.2 %) expected general practitioners (GPs) to be the best source of GDM information, following by diabetes educator nurses (45.9 %), diabetes support groups (33.9 %) and internet (32.1 %). However, women found that diabetes educator nurses were more helpful than GPs (32.6 and 20.2 %, respectively). Participants’ age and country of birth were statistically significant. For women aged over 30 years and women born overseas the internet was the most useful information source (68.9 and 77.1 % respectively). Overall, women were very satisfied (33.0 %) or satisfied (45.0 %) with how they were informed of the GDM diagnosis, although 26.0 % were informed by telephone and 16.0 % by text message. More than one-third (39.0 %) of women were not referred to sources of information by GPs at time of diagnosis of GDM (*p* <0.0001). Women who were referred reported that they were very satisfied (40.0 %) or satisfied (44.0 %) with information they received. Only 8.0 % of women reported dissatisfaction with the manner of health professionals.

**Conclusion:**

The results suggest that health professionals should be aware of the needs and expectations of women who have been diagnosed with GDM, with most women expecting to receive information on GDM from their GPs and diabetes educator nurses. The findings suggest that there is scope for improving how women are informed of the GDM diagnosis and given information, and in clinicians’ manner.

## Background

Gestational diabetes mellitus (GDM) is a condition that develops during pregnancy when the pancreas is not able to make enough insulin [1–3]. Although GDM usually resolves postpartum, there are long term consequences of obesity and risk of type 2 diabetes mellitus following diagnosis of GDM [4]. A recent study has found that women with a previous diagnosis of GDM carry a lifetime risk of progression to type 2 diabetes of up to 60 %. Thus, identification of those women at higher risk of progression to diabetes allows the timely introduction of measures to delay or prevent diabetes onset [5]. There are many different GDM diagnostic criteria in clinical use. In Australia, the 2-h pregnancy Oral Glucose Tolerance Test with 75 mg oral glucose load is used for diagnosis. Based on recent World Health Organization guidelines, GDM is diagnosed when fasting plasma glucose is between 5.1 and 6.9 mmol/L, or 1-h glucose is ≥10 mmol/L or 2-h glucose is between 8.5 and 11 mmol/L [6]. Previous research has shown women’s satisfaction with the diagnostic process [7], with women largely positive about their experiences of GDM diagnosis although believing the screening tests were not explained adequately [8].

Women newly diagnosed with GDM may seek information about the condition from many different sources [9], including health professionals (e.g., general practitioners, endocrinologists), books or magazines, internet [9], nurses and dieticians [10]. To date, however, there is limited research into women’s needs for information on GDM and their expectations of the most helpful and useful sources of information. Likewise, little is known about women’s satisfaction with the process of diagnosis and information provision.

Previous studies investigated women’s experience of GDM among different ethnic groups [11, 12] or were small studies or used qualitative methodologies [13–16]. Other studies focused on women’s experiences after diagnosis of GDM [7], the impact of diagnosis on women [7, 17, 18], or management of GDM [11]. Several researchers have looked at the use of internet as a source of information by pregnant women without GDM [19–25]. The aim of this study was to investigate the percentage of the needs and expectations of pregnant women with GDM about the best sources of information on GDM, and to evaluate their satisfaction with the diagnostic process and information provision.

## Methods

### Study design and setting

A quantitative study design was chosen and the study was approved by Western Health Human Research Ethics Committee (WH HREC), approval number HREC/11/WH/81. This study was carried out at the maternity diabetes clinic of Sunshine Hospital, Western Health, Victoria, Australia from December 2014 to May 2015. Informed written consent was obtained from each participant prior to enrolment.

### Study population and recruitment

#### Inclusion and exclusion criteria

Pregnant women aged 18–45 years, newly diagnosed with GDM and who attended the maternity diabetes clinic were invited to participate in the study. Pregnant women with pre-existing diabetes (types 1 and 2) and those who were unable to write and understand English were excluded.

#### Recruitment procedure

Eligible women who met the study criteria were invited to participate in the study and women who agreed to participate in this study were recruited until the required sample size is achieved. The recruitment of participants for this study was designed to use a consecutive sampling technique.

### Study tools and measurement

**Fig. 1:**
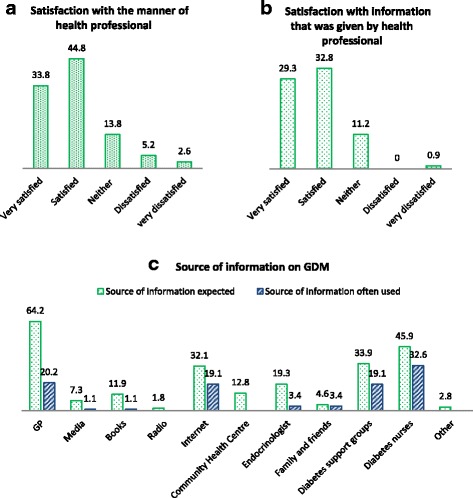
Satisfaction with the manner of health professional, satisfaction with information that was given by health professional and source of information on GDM

**Fig. 2:**
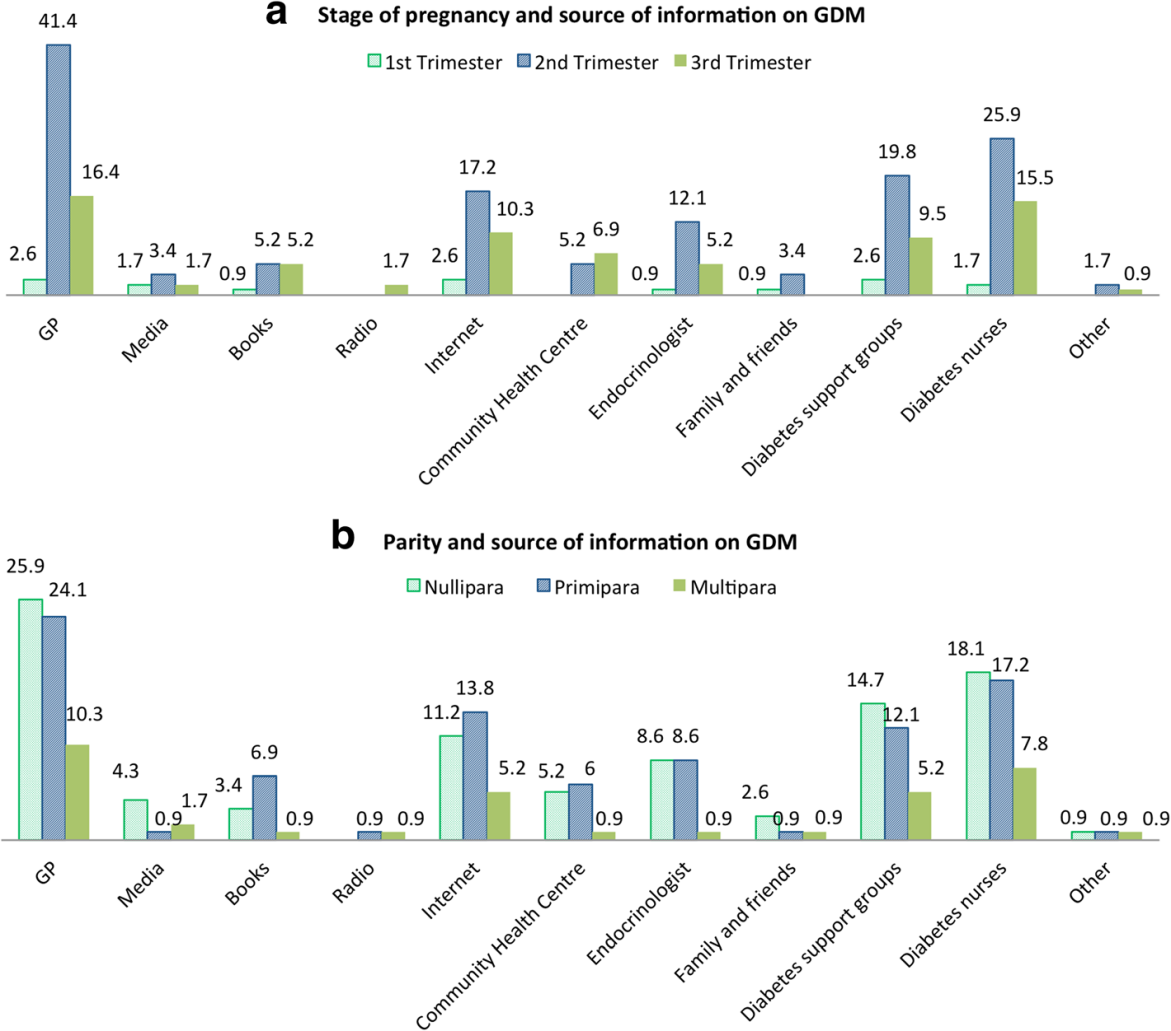
Stage of gestation, parity/number of birth and source of information on GDM needs

A self-administered written questionnaire was designed for the study. Questionnaire was developed from a previous study of Australian women [26] by the author and it was adapted to use in women with GDM. A pilot testing of the questionnaire was conducted to evaluate the validity and reliability of the questionnaire prior the study and it was approved by WH HREC. There were three opened-ended questions and 27 closed-ended questions, which included demographics information, maternal health, gestational diabetes, expected sources of information on GDM, satisfaction with the diagnostic process and satisfaction with health professionals at the time of the diagnosis. The required/expected source of information on GDM was also investigated through closed-ended questions that provided response options for women. For example, source of information on GDM was “general practitioner, magazine/media, book, Internet, etc.” (see Figs. 1c, 2a & 2b ). Five questions about satisfaction require participants to rate on a 5-point format scale (eg, very dissatisfied, dissatisfied, neither, satisfied and very satisfied). The three opened-ended questions require participants to identify a health professional that provided more information about GDM and identified the most useful health professional that women discussed concerns about GDM with. The questionnaire took 10–15 min to complete and women who agreed to participate were invited to complete the questionnaire at the clinic.

### Sample size

There were 280 women, who were newly diagnosed with GDM and attended the maternity diabetes clinic at Western Health in 2014 (2014 hospital data, data not published). The sample size was calculated by using the Raosoft sample size calculator (http://www.raosoft.com/samplesize.html) and in consultation with a biostatistician, the calculated sample size is 116 women with GDM, with a margin of error of 5 % and at 95 % confidence level with an 85 % response distribution in order to calculate the largest sample size needed.

### Data analysis

Analysis was performed using SPSS software (version 20.0; SPSS). Crosstabs, frequencies and descriptive statistics were used to summarize demographic data. Crosstabs with chi-squared test and the exact chi-squared were used to analyse data where appropriate. A probability of *p* ≤0.05 was considered statistically significant. Results were reported as frequencies and percentages.

## Results

### Response rate

The questionnaire was distributed to 130 women. Of these, five had pre-existing diabetes mellitus, two could not speak and write English, two did not complete the questionnaire, and five did not return completed questionnaires. In total, 116 questionnaires were completed, giving a response rate of 89.2 %.

### Demographic characteristics

The characteristics of the 116 participants are presented in Table 1. The mean age of participants was 31.7 years (range 19–43 years). The majority (70.7 %) of women were born outside Australia and used English as a second language. The most frequent religion was Christian (32.8 %). One-quarter (26.7 %) of women had secondary education only, 25.0 % had a tertiary certificate or diploma, 27.6 % had a bachelor degree, and 20.0 % had higher qualifications. One-third of women reported working full-time (33.6 %), 17.0 % worked part-time, while the remainder reported home duties (31.0 %), no paid work (9.5 %), or unemployed (7.8 %). The majority of women were married (76.7 %) and 46.6 % lived with their partners/husbands and children. Nearly half of women were nullipara (45.7 %), followed by primipara (33.6 %) and multipara (20.7 %).

**Table 1:** Demographic characteristics of women with GDM living in Australia

	*n* = 116	%
Age: Mean 31.7 years old, range (19 years – 43 years old):		
- 19–30 years	50	43.1
- >30 years	66	56.9
Country of birth:		
- Australia	34	29.3
- Overseas	82	70.7
Religion:		
- Christian	38	32.8
- Hindu	15	12.9
- Muslim/Islam	14	12.1
- No religion	25	21.6
- Other: Sikh, Buddhist, etc.	24	20.7
Education:		
- Year 12 or below	31	26.7
- Certificate, advanced diploma or diploma	29	25
- Bachelor degree	32	27.6
- Postgraduate degree (e.g. Graduate diploma, Masters or PhD)	24	20.7
Work status:		
- Full time	39	33.6
- Part time	20	17.2
- Other (eg, no paid work, unemployed)	57	49.1
Occupation:		
- Professional, high-level manager or administrator	15	12.9
- Manager or equivalent	12	10.3
- Clerical, service worker or sales	28	24.1
- Student (full-time)	5	4.3
- Home duties	36	31.0
- No paid work	11	9.5
- Other	9	7.8
Marital status:		
- Married	89	76.7
- Defacto	17	14.7
- Other	10	8.6
Household women live in:		
- Live alone	1	0.9
- Self and partner/husband only	39	33.6
- Self with partner/ husband and child or children	54	46.6
- Other	23	19.0
Parity/number of times that women have given birth:		
- Nullipara^a^	53	45.7
- Primipara^b^	39	33.6
- Multipara^c^	24	20.7

^a^Nullipara is a woman who has never given birth

^b^Primipara is a woman who is giving birth for the first time

^c^Multipara is a woman who has given birth two or more times

### Diagnosis of Gestational Diabetes Mellitus (GDM) and being informed of the diagnosis

The majority (63.8 %) of women were diagnosed with GDM in the second trimester of pregnancy, with mean gestational age 24.5 weeks (range 4–37 weeks). Most women (85.3 %) had seen only one health professional before the diagnosis of GDM was made but 14.7 % had seen more than one health professional. Nearly half the women (43.1 %) reported that they were informed of the diagnosis at the hospital clinic, 25.9 % were told in a telephone call, and 15.5 % received a text message. Only 9.5 % of women were informed of the diagnosis during an office visit consultation with a general practitioner (GP), and 6.0 % by a specialist.

The majority (61.2 %) of women were referred to sources of information at the time of diagnosis of GDM. Of the 61.2 %, the majority (46.5 %) of women reported that diabetes educator nurses provided more information about GDM, followed by GP (15.5 %), dietician (21.1 %), and a specialist oncologist (4.2 %). The majority of women also reported that they discussed questions/concerns about GDM with diabetes nurses (52.1 %), followed by GPs (17.0 %), oncologist (14.0 %), dietician (11.3 %), obstetrician (4.2 %), and none of the women reported discussion the information about GDM with midwife (Table 2).

**Table 2:** Health and diagnosis of GDM

	Frequency (*n* =116)	Percentage (%)
Gestational age at the time of diagnosis	Mean 24.5 weeks	Rage 4–37 weeks
Pregnancy status at the time of diagnosis		
- 1^st^ Trimester	6	5.2
- 2^nd^ Trimester	74	63.8
- 3^rd^ Trimester	36	31.0
Seeing >1 health professional before the diagnosis of GDM was made?		
- Yes	19	16.4
- No	97	83.6
Total:	116	100.0
Number of Health professional seen:		
- 1	99	85.3
- More than 1	17	14.7
- Total:	116	100.0
Type of Informed of the diagnosis of GDM:		
- At the hospital clinic	50	43.1
- By telephone	30	25.9
- During an office visit with GP	11	9.5
- During an office visit with the specialist	7	6.0
- Other (eg, text message)	18	15.5
Total	116	100.0
Refer to source of information at the time of diagnosis of GDM		
- Yes	71	61.2
- No	45	38.8
Total	116	100.0
Health professional that provided more information about GDM:		
- General practitioner	11	15.5
- Specialist endocrinologist	7	9.9
- Specialist obstetrician	3	4.2
- Diabetes educator nurses	33	46.5
- Dietician	15	21.1
- Midwife	1	1.4
- Other	1	1.4
Total:	71	100.0
The most useful health professional that women discussed questions about GDM with:		
- General practitioner	12	17.0
- Specialist endocrinologist	10	14.0
- Specialist obstetrician	3	4.2
- Diabetes educator nurses	37	52.1
- Dietician	8	11.3
- Midwife	0	0.0
- Other	1	1.4
Total:	71	100.0

### Satisfaction with health professionals

Women who were informed at the hospital clinic, by telephone call, or by text message reported that they were very satisfied (33.8 %) or satisfied (44.8 %) with the manner in which they were informed of the diagnosis, but a few were dissatisfied (5.2 %) or very dissatisfied (2.6 %) (Fig. 1a).

In comparison, satisfaction rates were much lower for women who were informed during GP consultations, with 3.5 % being very satisfied and 4.3 % satisfied with the manner in which they were informed of the diagnosis of GDM. Satisfaction rates were even lower for those who were informed by specialists, with 0.9 % being very satisfied and 2.6 % being satisfied (data not shown).

The women who were referred to sources of information at the time of diagnosis were very satisfied (29.3 %) or satisfied (32.8 %) with the information given by health professionals. Only 0.9 % reported very dissatisfied with the information that was provided at the hospital by health professionals (Fig. 1b).

### Required/expected sources of information and sources of information used most often

We investigated which sources of information women with GDM required or expected, and which ones were used most often. With multiple responses allowed, women reported that they required or expected information from their GPs (64.2 %), followed by diabetes educator nurses (45.9 %), diabetes support groups (33.9 %), internet (32.1 %), and endocrinologists (19.3 %) (Fig. 1c). The most helpful sources of information were diabetes educator nurses (32.6 %), followed by GPs (20.2 %), diabetes support group (19.1 %), and internet (19.1 %) (Fig. 1c).

### The relationship between demographic factors and the most useful source of information

We also investigated which sources of information were most useful to women of different age groups, country of birth, level of education and work status. For women aged 31 years and over, the most useful source of information was internet (68.6 %), followed by discussion with GP (62.9 %), and diabetes educator nurses (56.0 %). In women aged 19–30 years, 54.1 % found the diabetes support group was the most useful source. There were statistically significant differences between age of participants and most useful source of information for GPs (*p* = 0.017), diabetes support group (*p* = 0.003) and internet (*p* = 0.012) (Table 3).

**Table 3:** Demographic factors and the most useful sources of information from GP, Diabetes nurses, Diabetes support group and internet

	GP			Diabetes Nurses			Diabetes Support Group			Internet		
	*n* = 70^a^	%	*p*	*n* = 50^b^	%	*p*	*n* = 37^c^	%	*p*	*n* = 35^d^	%	*p*
Age:												
- 19–30 years	26	37.1	0.017*	22	44.0	0.743	20	54.1	0.003*	11	31.4	0.001*
- > 30 years	44	62.9		28	56.0		17	45.9		24	68.6	
Country of birth:												
- Australia	22	31.4	0.182	18	36.0	0.237	16	43.2	0.002*	8	22.9	0.031*
- Overseas	48	68.6		32	64.0		21	56.8		27	77.1	
Education:												
- Year 12 or below	20	28.6	0.138	9	18.0	0.970	8	21.6	0.630	9	25.7	0.268
- Certificate, or diploma	17	24.2		15	30.0		12	32.4		8	22.9	
- Bachelor degree	23	32.9		16	32.0		10	27.0		10	28.5	
- Postgraduate degree	10	14.3		10	20.0		7	19.0		8	22.9	
Work status:												
- Full time	24	34.3	0.876	21	42.0	0.507	12	32.4	0.930	12	34.2	0.262
- Part time	13	18.6		7	14.0		7	19.0		8	22.9	
- Other	33	47.1		22	44.0		18	48.6		15	42.9	

Multiple response crosstabs was used. Kruskal-Wallis test, *T*-test and Chi-Square test for *p* value

^a,b,c,d^The number in each category who expected information source from GP^a^, diabetes nurses^b^, diabetes support group^c^ and the internet^d^

*P <0.05 was considered statistically significant

For women born outside Australia, the most useful source of information was internet (77.1 %), followed by information from GP (68.6 %), diabetes educator nurses (64.0 %) and diabetes support group (56.8 %). There were statistically significant differences between country of birth for internet (*p* = 0.031) and diabetes support group (*p* = 0.002) (Table 3).

Women who had completed undergraduate degrees reported GPs (32.9 %), diabetes educator nurses (32.0 %), internet (28.8 %), and diabetes support group (27.0 %) as the most useful sources, but the differences were not statistically significant.

Women who worked full-time reported that diabetes educator nurses were the most useful source (42.0 %) while women who were not in the paid workforce identified diabetes support groups as the most useful source (48.6 %), followed by GPs (47.1 %), diabetes educator nurses (44.0 %), and internet (42.9 %). None of these differences was statistically significant (Table 3).

### Stage of gestation, parity/number of birth and the need for sources of information on GDM

The relationship between stage of pregnancy, parity and source of information needs is shown in Fig. 2. Women in second trimester required more sources of information than women in first and third trimesters. The majority of women who were pregnant in second trimester used GPs (41.4 %) as the major source of information during pregnancy, followed by diabetes educator nurses (17.2 %), diabetes support group (19.8 %) and internet (17.2 %). Only 16.4 % of pregnant women in the third trimester required information from GPs, followed by diabetes nurses (15.5 %) and internet (10.3 %) (Fig. 2a).

Nulliparous women (or women who has never given birth) required more source of information from GPs (25.9 %) compared to primiparous women (24.1 %) and multiparous women (10.3 %), followed by diabetes nurses (18.1, 17.2 and 7.8 %, respectively), and diabetes support group (14.7, 12.1 and 5.2 %). Whereas, 13.8 % of primiparous women reported that they needed more information from Internet compared to nulliparous women (11.2 %) and multiparous women (5.2 %) (Fig. 2b).

## Discussion

Patient satisfaction and adequate sources of information provided by health professionals may help improve health recovery and prevent complications from GDM. This study evaluated women’s satisfaction with the diagnostic process for GDM, provision of information, and satisfaction with sources of information recommended by health professionals. Overall, we found that the majority of women were very satisfied or satisfied with the manner in which they were informed of the diagnosis, although more than one-third of women were not referred to sources of information at the time of diagnosis of GDM. There were significant differences between younger and older women, and women born in Australia and overseas, as to the most useful sources of information. This study also investigated women’s needs and expectations about the best sources of information on GDM. Most women expected to receive advice and diabetic information from their GPs but this did not eventuate and instead women sought information from diabetes educator nurses.

When newly diagnosed with GDM, some women find the necessary dietary and lifestyle changes challenging and difficult [27, 28]. Most women in the current study were very satisfied or satisfied with the manner in which they were informed of the diagnosis. This supported a previous study in Australia which reported that the majority (83 %) of women with GDM were very satisfied or satisfied with the diagnostic process and manner in which they were informed of the diagnosis [8].

The findings of this study suggest it is difficult for women with GDM to discuss information about the disease with their health professionals at the time of diagnosis and they may not be given adequate information regarding GDM. Future research needs to focus on clinician-patient communication during doctors’ visit, because communication problems are a common reason for patient dissatisfaction during consultations with doctors or specialists [29]. The present study found that while the majority (62.1 %) of women were very satisfied or satisfied with information provided at the time of GDM diagnosis, one-third of women felt they were not given adequate information. A few were dissatisfied or very dissatisfied with the information given, which may have contributed to a perceived negative experience [29].

The findings from this study support the statement that women access information from a variety of sources [25]. We found that women identified diabetes educator nurses as the most useful source of information during pregnancy, followed by discussion with GPs, and that they frequently sought information from the internet. A previous study has reported that women prefer receiving information from healthcare professionals, for whom provision of health information is a significant part of their role [30]. Interestingly, no respondents in the present study identified discussions with midwives. This contrasts with the study by Grimes et al. of sources of information used by Australian women during pregnancy which reported midwives as the most commonly used and most useful source of information [25]. The reason for this difference may be that Grimes et al. studied women experiencing normal pregnancies, whereas in the present study participants were diagnosed with GDM and hence there was greater emphasis on using diabetes educator nurses to manage the gestational diabetes. To date, no other study has examined sources of information about GDM used by pregnant women diagnosed with GDM, and in particular their expectations about where to find the most useful information.

Although discussion with diabetes educator nurses was identified as the source of information used most often by pregnant women in this study, the majority of women also required and expected to receive more information from GPs during hospital or office visits. Surprisingly, the study found that one-third of participants received no referral to sources of information when given their diagnosis. This may be a source of dissatisfaction with the manner of health professionals and the information given at the time of diagnosis. This finding has implications for clinical practice because it may raise awareness among clinicians about women’s needs for knowledge and information to promote healthy outcomes.

Women in the present study had complex pregnancies and were at high risk of developing type 2 diabetes later in life. Because of this, their information needs were higher than those of women with normal pregnancies. Thus, healthcare providers may have different opportunities to address information needs. Diabetes educator nurses may be able to give women more opportunity to talk about their concerns and have their questions answered during appointments, and this may be related to time constraints, different practices or women’s own perceptions of the roles of providers. This view is supported by a study on the information needs of first-time pregnant women in the United Kingdom that suggested that when women said “they wanted more information”, what they really wanted was an opportunity to voice their concerns and have their questions answered by supportive carers [31].

Previous studies have reported that women access the internet for support and pregnancy-related information to assist in their decision-making [20, 24, 32] and they use the internet in response to inadequate information provision by health professionals [24]. The growth in internet availability over the past decade has given many women access to a broad range of information on pregnancy, birth, and parenting. Women may prefer the internet to other sources of information because of privacy, accessibility and scope of information available [20, 33, 34]. Sociodemographic factors may influence which women use the internet for health information. In the present study, it was older women and those born outside Australia who turned to the internet. These women may believe the internet gives greater privacy and provides more accessible information than face-to-face discussions with health professionals. Women whose first language is not English may find information that is easier to understand online, especially if it is in their first language. These findings support previous studies [20, 33, 34]. Other sociodemographic variables such as level of education or work status were not associated with an increased likelihood of citing the internet as the most useful source of information on GDM during pregnancy. This contrasts with previous studies showing that women who had higher levels of education were the group most likely to seek online health information [34–36]. Further research is needed to clarify this question.

### Limitations and strengths

The present study has some limitations. It was conducted in one location and results cannot be generalized. The findings may not fully reveal the experiences of women in regional or rural centres, or other countries who may not have access to all the sources of information offered at the study site.

Quantitative studies might not demonstrate the depth of data related to the experience of diagnosis and qualitative research would add to this description. Further qualitative research in this area may provide a more complete understanding about the ways in which individuals seek information and the impact of not having their information needs met.

The current study did not explore the content information provided by the health professionals. Future study is needed to address this area and conducting an in-depth interview may be useful to obtain detailed information about the topic.

The strengths of the study include its multicultural sample of women living in Australia, the relatively high response rate, and the fact that it was performed during pregnancy, thereby reducing recall bias and eliminating bias related to events at the time of delivery. Our findings can be added to the literature on what is currently known about this topic.

### Implications

Implications for practice and future research have been suggested in the discussion. This study highlights the need for health professionals and maternity service providers to sustain, or to recommend, multiple sources of information for use by women diagnosed with GDM. Findings suggest that GPs neither provided information nor referred women to sources of information at the time of the diagnosis. This highlights an important point for clinical practice because it may help to raise clinicians’ awareness of this issue and therefore provide women with the knowledge and information they need to promote healthy outcome. General practitioners should identify sources of information for women at the time of diagnosis. The use of telephone calls or text messages to inform women of their diagnosis should be avoided to minimise women’s dissatisfaction with the manner in which they were informed.

It appears that women benefit from access to diabetes educators during their pregnancies by having their information needs met. Discussion with a diabetes educator was important and the most useful source of information for the majority of women in this study. It may be beneficial if women who require medical care throughout their pregnancy are also able to access a diabetes educator nurse for discussion of their information needs about GDM during pregnancy.

## Conclusion

The quality of clinician-patient communication is a critical factor influencing treatment outcomes and patient satisfaction with care. The key findings of this study build on what is known about information provision to pregnant women with GDM. Women with GDM revealed that they expected to receive information about GDM from their GPs. The present study showed that GPs did not refer women to information about GDM at the time of diagnosis. Thus, women turned for information to diabetes nurses and diabetes support groups. Health professionals should be aware of the needs and expectation of women who have been diagnosed with GDM, because the results of this study suggest that most women expect to receive information on GDM from their GPs and diabetes nurses. The findings suggest that there is scope for improvement in information provision, conveying the diagnosis and manner clinicians’ manner.

## Abbreviations

DM: Diabetes mellitus
GDM: Gestational diabetes mellitus
GP: General practitioner
WH HREC: Western Health Human Research Committee
